# Antimicrobial sensitivity patterns of cerebrospinal fluid (CSF) isolates in Namibia: implications for empirical antibiotic treatment of meningitis

**DOI:** 10.1186/2052-3211-6-4

**Published:** 2013-06-13

**Authors:** Assegid Mengistu, Johannes Gaeseb, Gottfried Uaaka, Christophine Ndjavera, Kennedy Kambyambya, Lazarus Indongo, Francis Kalemeera, Christopher Ntege, David Mabirizi, Mohan P Joshi, Evans Sagwa

**Affiliations:** 1 Ministry of Health and Social Services, Windhoek, Namibia; 2 Namibia Institute for Pathology, Windhoek, Namibia; 3 Systems for Improved Access to Pharmaceuticals and Services, implemented by Management Sciences for Health, Windhoek, Namibia

**Keywords:** Celebrospinal fluid, Antimicrobial resistance, Culture and sensitivity, Empiric therapy, Meningitis, Namibia

## Abstract

**Objective:**

Bacterial meningitis is a medical emergency associated with high mortality rates. Cerebrospinal fluid (CSF) culture is the “gold standard” for diagnosis of meningitis and it is important to establish the susceptibility of the causative microorganism to rationalize treatment. The Namibia Standard Treatment Guidelines (STGs) recommends initiation of empirical antibiotic treatment in patients with signs and symptoms of meningitis after taking a CSF sample for culture and sensitivity. The objective of this study was to assess the antimicrobial sensitivity patterns of microorganisms isolated from CSF to antibiotics commonly used in the empirical treatment of suspected bacterial meningitis in Namibia.

**Methods:**

This was a cross-sectional descriptive study of routinely collected antibiotic susceptibility data from the Namibia Institute of Pathology (NIP) database. Results of CSF culture and sensitivity from January 1, 2009 to May 31, 2012, from 33 state hospitals throughout Namibia were analysed.

**Results:**

The most common pathogens isolated were *Streptococcus species, Neisseria meningitidis*, *Haemophilus influenzae*, *Staphylococcus*, and *Escherichia coli*. The common isolates from CSF showed high resistance (34.3% –73.5%) to penicillin*.* Over one third (34.3%) of *Streptococcus* were resistance to penicillin which was higher than 24.8% resistance in the United States. *Meningococci* were susceptible to several antimicrobial agents including penicillin. The sensitivity to cephalosporins remained high for *Streptococcus*, *Neisseria*, *E. coli* and *Haemophilus*. The highest percentage of resistance to cephalosporins was seen among ESBL *K. pneumoniae* (n = 7, 71%–100%), other *Klebsiella* species (n = 7, 28%–80%), and *Staphylococcus* (n = 36, 25%–40%).

**Conclusions:**

The common organisms isolated from CSF were *Streptococcus Pneumoniae*, *Neisseria meningitidis*, *Haemophilus influenzae*, *Staphylococcus*, and *E. coli*. All common organisms isolated from CSF showed high sensitivity to cephalosporins used in the empirical treatment of meningitis. The resistance of the common isolates to penicillin is high*.* Most ESBL *K. pneumoniae* were isolated from CSF samples drawn from neonates and were found to be resistant to the antibiotics recommended in the Namibia STGs. Based on the above findings, it is recommended to use a combination of aminoglycoside and third-generation cephalosporin to treat non–ESBL *Klebsiella* isolates. Carbapenems (e.g., meropenem) and piperacillin/tazobactam should be considered for treating severely ill patients with suspected ESBL *Klebsiella* infection. Namibia should have a national antimicrobial resistance surveillance system for early detection of antibiotics that may no longer be effective in treating meningitis and other life-threatening infections due to resistance.

## Introduction

Bacterial meningitis is an acute infection in which the meninges, the subarachnoid space, and the brain parenchyma are all frequently involved in the inflammatory reaction. This disease is characterized by severe headache, fever, intolerance to light and sound and rigidity of muscles, especially those of the neck. The central nervous system (CNS) inflammatory reaction from bacterial meningitis may result in decreased consciousness, seizures, raised intracranial pressure, and stroke [1].

Bacterial meningitis is a medical emergency. The therapeutic goal is to initiate antibiotic therapy within 60 minutes of a patient’s arrival in the emergency room. In patients suspected of having bacterial meningitis, cerebrospinal fluid (CSF) should be obtained for cultures and empirical antimicrobial therapy initiated without delay [2,3]. Diagnosis of bacterial meningitis is confirmed by CSF culture the “gold standard” for diagnosis of meningitis and it is equally important to obtain the antimicrobial susceptibility of the causative microorganism to rationalize treatment [4,5].

The organisms most commonly responsible for community-acquired bacterial meningitis are *Streptococcus pneumoniae*, *Neisseria meningitidis*, *Streptococci* group B, *Listeria monocytogenes,* and *Haemophilus influenza*[6,7]. In children, meningococcal, *Haemophilus influenzae* type b (Hib), and pneumococcal infections are the most common causes [7].

Specific antibiotic treatment for bacterial meningitis depends upon identification of the causative organism. More than 80% of patients with common bacterial meningitis are culture positive. However, CSF culture positivity decreases with prior antibiotic treatment before lumbar puncture [4].

As infections of the CNS are potentially life threatening, empirical therapy should be initiated promptly whenever bacterial meningitis is a major diagnostic consideration. The Namibia Standard Treatment Guidelines (STGs) recommend initiation of empirical antibiotics treatment in patients with signs and symptoms of meningitis after taking a CSF sample for analysis, culture, and antibiotic sensitivity testing. The preferred medicines for patient with signs and symptoms of meningitis are based on knowledge of the common causative agent of meningitis in the specific patient group and the sensitivity of the suspected pathogen to the commonly used antimicrobials.

However, many infectious diseases are becoming increasingly difficult to treat because of antimicrobial-resistant organisms. Both the epidemiology of bacterial meningitis and the sensitivity to antibiotics are changing as a result of the widespread use of antimicrobials and other factors [4]. Antimicrobial susceptibility data among CNS pathogens is therefore important to effectively manage meningitis patients in the first critical hours of their treatment [8]. Empirical antibiotic therapy should be adjusted to local drug resistance patterns and clinical subgroups. Accurate information regarding the important etiological agents and populations at risk is necessary to ascertain public health measures and ensure appropriate management of bacterial meningitis [9,10].

It is therefore essential to monitor the emergence of resistance to antibiotics that are used for the empirical treatment as delay in providing effective treatment may adversely affect a patient’s treatment outcome.

### Objectives

• To determine the common microorganisms isolated from cerebrospinal fluid that are responsible for infectious meningitis in Namibia

• To establish the prevalence of antimicrobial sensitivity of microorganisms isolated from clinical cerebrospinal fluid samples in Namibia

• To determine the most appropriate medicines for the treatment of meningitis in Namibia

## Methods

This was a cross-sectional descriptive study using routinely collected antibiotic susceptibility data from Namibia Institute of Pathology (NIP) database. Results of CSF culture and sensitivity from January 1, 2009 to May 31, 2012, from 33 out of 35 state hospitals throughout Namibia were stored in Meditech®. NIP uses this commercial laboratory management software to capture routinely collected antibiotic susceptibility and other laboratory data from public sector health facilities throughout the country. Cerebrospinal fluid clinical samples were transported and immediately processed at the NIP’s regional or central laboratory. Standard CSF bacterial culture is accomplished using horse blood agar (5%) or chocolate agar.

Pathogen identification and antibacterial susceptibilities were performed as appropriate using Wellcogen® Bacterial Antigen Kit, Gram stain, and methylene blue stain. Standard antibiogram profile tests were done for specific microorganism isolates with some variations according to the doctors’ request. Culture results were read after 24 hours of incubation. Plates were re-incubated for a further 24 hours and re-examined for additional organisms. The results were recorded on a worksheet and entered into Meditech. Printed results were sent back to the clinicians who could also obtain the results by logging into the tool’s web-based reporting module.

WHONET, a database software developed by the World Health Organization Collaborating Centre for Surveillance of Antimicrobial Resistance, was used to extract the data from all CSF samples. The extracted data was cleaned thorough visual checks and preliminary frequency counts on the raw data set and identified errors were corrected by the lead author. Cleaned data was analysed using Statistical Package for the Social Sciences® version 12.0.1.

The main outcome variable was the proportion of samples with positive cultures of suspected microorganisms. We also looked into the sensitivity patterns of isolates from CSF. Descriptive statistics were used to summarize the frequencies and distributions of microbial isolates and their sensitivity to various antimicrobials.

Since the analysis was conducted on de-identified electronic records of samples tested by the NIP, individual patient consent was not required. Authorization to conduct the analysis was granted by the Permanent Secretary of Ministry of Health and Social Services of Namibia (MoHSS) and the NIP management.

## Results

A total of 7,267 CSF samples were tested at the NIP central and regional laboratories for culture and antimicrobial sensitivity. Out of the 7,267 CSF samples submitted, 701 (9.6%) showed growth of microorganisms. Out of the 701 samples with growth of microorganism, 503 (71.8%) grew bacteria, 188 (26.8%) grew fungi (mainly *Cryptococcus*), and the remaining 10 samples (1.4%) grew mycobacterium (Table 1).

**Table 1:** Type of organism isolated from CSF

**Type of organism**	**Frequency (%)**	**Cumulative (%)**
Gram Negative	239 (3.3%)	239 (3.3%)
Gram Positive	264 (3.6%)	503 (6.9%)
Fungi	188 (2.6%)	691 (9.5%)
Mycobacterium	10 (0.1%)	701 (9.6%)
No growth	6,566 (90.4%)	7,267 (100.0%)
Total	7267 (100.0%)	

Source: MEDITECH Database 2009–2012.

The most frequent gram positive organisms isolated were *Streptococcus species* (n = 206, 40.9%), *Staphylococcus* (n = 36, 7.2%), and *Enterococcus species* (n = 9, 1.8%). The three most frequently identified gram negative organisms isolated were *Neisseria meningitidis* (*N. meningitidis*) (n = 107, 21.3%), *Haemophilus influenzae* (n = 59, 11.7%), and *Escherichia coli* (*E. coli*) (n = 22, 4.4%) (Table 2). 

**Table 2:** Bacteria isolated from CSF

**Organism isolate**	**Frequency of isolate**	**%**	**Cumulative frequency**	**Cumulative %**
Streptococcus pneumoniae	187	37.18	187	37.18
Neisseria meningitids group A	53	10.54	240	47.71
Neisseria meningitidis	48	9.54	288	57.26
Haemophilus influenzae	38	7.55	326	64.81
Escherichia coli	22	4.37	348	69.18
Staphylococcus aureus	19	3.78	367	72.96
Haemophilus species	15	2.98	382	75.94
Staphylococcus epidermides	10	1.99	392	77.93
Streptococcus species	10	1.99	402	79.92
Enterococcus faecalis	7	1.39	409	81.31
ESBL Klebsiella pneumoniae	7	1.39	416	82.7
Haemophilus influenzae type B	6	1.19	422	83.9
Pseudomonas aeruginosa	5	0.99	427	84.89
Streptococcus group B	4	0.8	431	85.69
Acinetobacter baumanii	4	0.8	435	86.48
Klebsiella pneumoniae	4	0.8	439	87.28
Bacillus cereus	3	0.6	442	87.87
Micrococcus species	3	0.6	445	88.47
Gram negative bacilli	3	0.6	448	89.07
Serratia liquifaciens	3	0.6	451	89.66
Gram positive cocci	2	0.4	453	90.06
Listeria monocygenes	2	0.4	455	90.46
Staphylococcus capitis	2	0.4	457	90.85
Staphylococcus haemolyticus	2	0.4	459	91.25
Streptococcus group A	2	0.4	461	91.65
Streptococcus viridans group	2	0.4	463	92.05
Acinetobacter lwoffii	2	0.4	465	92.45
ESBL’s Escherichia coli	2	0.4	467	92.84
Klebsiella oxytoca	2	0.4	469	93.24
Neisseria meningitidis group C	2	0.4	471	93.64
Neisseria meningitidis W135	2	0.4	473	94.04
Neisseria species	2	0.4	475	94.43
Salmonella species	2	0.4	477	94.83
Serratia marcescens	2	0.4	479	95.23
Sphingomonas paucimobilis	2	0.4	481	95.63
Bacillus species	1	0.2	482	95.83
Diphtheroid organisms	1	0.2	483	96.02
Enterococcus faecium	1	0.2	484	96.22
Enterococcus species	1	0.2	485	96.42
Kocuria varians	1	0.2	486	96.62
Staphylococcus hominis	1	0.2	487	96.82
Staphylococcus saprophyticus	1	0.2	488	97.02
Staphylococcus simulans	1	0.2	489	97.22
Streptococcus agalacteae	1	0.2	490	97.42
Acinetobacter junii	1	0.2	491	97.61
Acinetobacter species	1	0.2	492	97.81
Aeromonas hydrophilia	1	0.2	493	98.01
Coliform bacillus	1	0.2	494	98.21
Enterobacter species	1	0.2	495	98.41
Haemophilus parainfluenzae	1	0.2	496	98.61
Klebsiella ozaenae	1	0.2	497	98.81
Moraxella species	1	0.2	498	99.01
Morganella morganii	1	0.2	499	99.2
Pseudomonas paucimobilis	1	0.2	500	99.4
Pseudomonas species	1	0.2	501	99.6
Salmonella omnivalent positive	1	0.2	502	99.8
Yersinia pestis	1	0.2	503	100

Source: MEDITECH Database 2009–2012.

Extended spectrum beta lactamase (ESBL) *Klebsiella pneumoniae* (*K. pneumonia*) was the most frequently isolated microorganism in CSF samples collected from neonates (4/9) whereas *Haemophilus, Streptococcus,* and *Staphylococcus* were common in CSF samples collected from infants aged from one to 11 months. In the age group 1–5 years, *Neisseria, Haemophilus,* and *Streptococcus* were commonly isolated (Table 3). *Neisseria* and *Streptococcus* were commonly isolated in CSF samples drawn from the age group 6–12 years. In adults and adolescents 12 years and older, *Neisseria, E. coli, Streptococcus,* and *Staphylococcus* were the most frequent isolates (Table 3). The majority of the isolated bacteria (n = 409, 81.3%) were from inpatient CSF samples, followed by samples from the outpatient (n = 40, 8.0%) and paediatrics departments (n = 43, 8.5%) (Table 4).

**Table 3:** Frequency of organisms isolates by age group

**Organism**	**<1 mo**	**1-11 mos**	**1-55 yrs**	**6-12 yrs**	**> 12 yrs**	**Age Unk**	**Total**	**% of total**
Streptococcus pneumoniae	2	31	27	17	87	42	187	37.18
Neisseria species	2	5	14	18	56	12	107	21.27
Haemophilus species	0	18	24	2	9	6	59	11.73
Staphylococcus	0	10	6	2	11	7	36	7.16
Others Gram negative	0	11	2	0	7	2	22	4.39
Escherichia coli	1	2	1	0	15	3	22	4.37
Streptococcus species							19	3.77
Other Gram positives	0	4	3	1	5	0	13	2.6
Enterococcus species	0	3	0	2	1	3	9	1.79
Acinetobacter species	0	4	0	2	2	0	8	1.59
ESBL’s Klebsiella pneumoniae	4	2	0	0	0	1	7	1.39
Klebsiella	0	1	1	0	4	1	7	1.39
Pseudomonas species	0	2	2	1	2	0	7	1.39
Total	9	93	80	45	199	77	503	100.02

Source: MEDITECH Database 2009–2012. Legend: mo=month; mos=months; yrs=years; age unk= age unknown; % = percent.

**Table 4:** Frequency of organisms isolated by hospital department

**Organism**	**Inpatient**	**Outpatient**	**Pediatrics**	**Missing**	**Total**
Streptococcus species	162	16	19	9	206
Neisseria species	94	11	2	0	107
Haemophilus species	47	2	9	1	59
Staphylococcus aureus	34	1	1	0	36
Escherichia coli	13	7	1	1	22
Other Gram negative	20	2	0	0	22
Other Gram positive	12	0	1	0	13
Enterococcus species	8	0	1	0	9
Acinetobacter species	6	0	2	0	8
ESBL’s Klebsiella pneumoniae	6	1	0	0	7
Klebsiella	7	0	0	0	7
Pseudomonas species	0	0	7	0	7
Total	409 (81.3%)	40 (8.0%)	43 (8.5%)	11 (2.2%)	503 (100%)

Source: MEDITECH Database 2009–2012.

We found that *Streptococcus species* were the most common gram positive organisms isolated from clinical CSF samples analyzed by NIP. *Streptococci* showed very high resistance to sulfamethoxazole/trimethoprim (n = 125, 71.4%) and high resistance to oxacillin (n = 69, 45.1%), penicillin (n = 58, 34.3%), and amoxicillin (n = 6, 25.0%). *Streptococci* were moderately resistant for gentamicin, tetracycline, clindamycin, and erythromycin, ranging from 12.4 to 20.0%. The resistance to ceftriaxone, ofloxacin, cefuroxime, chloramphenicol, and vancomycin ranged from 2.2% to 5.5%. All of the bacterial isolates (n = 34, 100.0%) that were tested for ciprofloxacin were found to be sensitive to this medicine (Table 5).

**Table 5:** Susceptibility of organisms isolated from CSF

**Organism**	**Antimicrobial**	**Total, N**	**S**	**S, %**	**R**	**R, %**	**I**	**I, %**
Streptococcus 206 isolates	Ciprofloxacin	34	34	100	0	0		0
	Ceftriaxone	89	87	97.8	2	2.2	0	0
	Ofloxacin	40	39	97.5	1	2.5	0	0
	Cefuroxime	28	27	96.4	1	3.6	0	0
	Chloramphenicol	171	160	93.6	9	5.3	2	1.2
	Vancomycin	110	102	92.7	6	5.5	2	1.8
	Erythromycin	186	162	87.1	23	12.4	1	0.5
	Clindamycin	140	120	85.7	19	13.6	1	0.7
	Tetracycline	180	146	81.1	25	13.9	9	5
	Gentamicin	5	4	80	1	20	0	0
	Amoxicillin	24	18	75	6	25	0	0
	Penicillin	169	109	64.5	58	34.3	2	1.2
	Oxacillin	153	79	51.6	69	45.1	5	3.3
	Sulfamethoxazole/trimethoprim	175	48	27.4	125	71.4	2	1.1
Neisseria meningitidis 107 isolates	Chloramphenicol	102	97	95.1	3	2.9	2	2
	Ceftriaxone	105	97	92.4	5	4.8	3	2.9
	Penicillin	105	86	81.9	16	15.2	3	2.9
	Erythromycin	14	10	71.4	4	28.6	0	0
	Tetracycline	24	17	70.8	3	12.5	4	16.7
	Sulfamethoxazole/trimethoprim	102	22	21.6	79	77.5	1	1
Haemophilus 59 isolates	Amoxicillin	53	32	60.4	18	34	3	5.7
	Amoxicillin/Clavulanic Acid	18	14	77.8	4	22.2		0
	Ceftriaxone	52	46	88.5	5	9.6	1	1.9
	Cefuroxime	20	14	70	5	25	1	5
	Chloramphenicol	55	48	87.3	6	10.9	1	1.8
	Ciprofloxacin	11	11	100	0	0		0
	Ofloxacin	23	22	95.7	0	0	1	4.3
	Penicillin	6	6	100		0		0
	Tetracycline	27	21	77.8	4	14.8	2	7.4
	Sulfamethoxazole/trimethoprim	37	10	27	25	67.6	2	5.4
Staphylococcus 36 isolates	Amikacin	7	7	0	0	0	0	0
	Vancomycin	22	20	90.9	0	0	2	9.1
	Fusidic acid	34	29	85.3	1	2.9	4	11.8
	Ciprofloxacin	21	17	81	4	19		0
	Cephalothin	8	6	75	2	25	0	0
	Clindamycin	27	19	70.4	6	22.2	2	7.4
	Tetracycline	27	19	70.4	8	29.6		0
	Erythromycin	31	21	67.7	10	32.3	0	0
	Oxacillin	9	6	66.7	3	33.3	0	0
	Cloxacillin	29	19	65.5	10	34.5		0
	Cefuroxime	5	3	60	2	40	0	0
	Gentamicin	17	7	41.2	9	52.9	1	5.9
	Penicillin	34	8	23.5	25	73.5	1	2.9
	Amoxicillin	14	3	21.4	11	78.6	0	0
	Sulfamethoxazole/trimethoprim	29	0	0	18	62.1	11	37.9
Escherichia coli 22 isolates	Ceftriaxone	17	17	100		0		0
	Chloramphenicol	6	6	100		0		0
	Ciprofloxacin	13	12	92.3	1	7.7		0
	Cefuroxime	19	15	78.9	2	10.5	2	10.5
	Piperacillin-Tazobactam	4	3	75	1	25		0
	Cephalothin	10	7	70	1	10	2	20
	Gentamicin	17	11	64.71	3	17.65	3	17.65
	Tetracycline	5	3	60	2	40		0
	Amoxicillin/Clavulanic Acid	7	4	57.14	3	42.86		0.0
	Erythromycin	4	2	50	2	50		0
	Penicillin	4	2	50	2	50		0
	Sulfamethoxazole/trimethoprim	21	3	14.29	18	85.71		0
	Amoxicillin	22	3	13.6	19	86.4		0
Enterococcus species 9 isolates	Penicillin	8	7	87.5	1	12.5	0	0
	Vancomycin	9	7	77.8	1	11.1	1	11.1
	Amoxicillin	7	5	71.4	2	28.6	0	0
	Ciprofloxacin	3	2	66.7	1	33.3		0
	Erythromycin	3	1	33.3	2	66.7	0	0
	Clindamycin	8	2	25	6	75	0	0
	Tetracycline	2	0	0	2	100	0	0
	Sulfamethoxazole/trimethoprim	2	0	0	2	100	0	0
Acinetobacter species 8 isolates	Imipenem	8	7	87.5	1	12.5		0
	Sulfamethoxazole/trimethoprim	7	6	85.7	1	14.3		0
	Gentamycin	8	6	75	2	25	0	0
	Piperacillin-Tazobactam	8	6	75	1	12.5	1	12.5
	Ceftazidime	6	4	66.7	1	16.7	1	16.7
	Piperacillin	2	1	50	1	50		0
Klebsiella 7 isolates	Ciprofloxacin	5	5	100		0		0
	Amikacin	6	5	83.3	1	16.7		0
	Ceftriaxone	7	5	71.4	2	28.6		0
	Gentamicin	6	4	66.7	1	16.7	1	16.7
	Piperacillin-Tazobactam	3	2	66.7	1	33.3		0
	Cefuroxime	6	3	50	2	33.3	1	16.7
	Sulfamethoxazole/trimethoprim	7	3	42.9	4	57.1		0
	Cephalothin	5	1	20	4	80	0	0
	Amoxicillin	7	1	14.3	4	57.1	2	28.6
ESBL’s Klebsiella pneumoniae 7 isolates	Amikacin	7	5	71.43	1	14.3	1	14.3
	Ciprofloxacin	7	7	100		0		0
	Imipenem	7	7	100		0		0
	Piperacillin-Tazobactam	7	7	100		0		0
	Ceftazidime	7	2	28.6	5	71.4		0
	Amoxicillin	7		0	7	100		0
	Ceftriaxone	7		0	7	100		0
	Cefuroxime	7		0	7	100		0
	Gentamicin	7		0	7	100		0
	Sulfamethoxazole/trimethoprim	7		0	7	100		0
Pseudomonas species 7 isolates	Amikacin	7	7	100		0		0
	Ceftazidime	7	7	100		0		0
	Ciprofloxacin	6	6	100		0		0
	Imipenem	6	6	100		0		0
	Gentamycin	6	5	83.3	1	16.7	0	0
	Piperacillin-Tazobactam	7	4	57.1		0	3	42.9
	Sulfamethoxazole/trimethoprim	7	3	42.9	4	57.1		0

Source: MEDITECH Database 2009–2012. legend: S=sensitive; R=resistant and I=indeterminate.

Among the gram negative bacteria, *N.meningitidis* was the most frequently isolated pathogen, and showed the following patterns of resistance sulfamethoxazole/trimethoprim (n = 79, 77.5%), erythromycin (n = 4, 28.6%), penicillin (n = 16, 15.2%), tetracycline (n = 3, 12.5%), cefuroxime (n = 5, 4.8%), and chloramphenicol (n = 3, 2.9%) (Table 5).

*Haemophilus* isolates were resistant to sulfamethoxazole/trimethoprim (n = 25, 67.6%), amoxicillin (n = 18, 34%), cefuroxime (n = 5, 25%), amoxicillin/clavulanic acid (n = 4, 22.2%), tetracycline (n = 4, 14.8%), chloramphenicol (n = 6, 10.9%), and ceftriaxone (n = 5, 9.6%). All *Haemophilus* isolates were sensitive to penicillin G and ciprofloxacin (Table 5).

*Staphylococcus* isolates showed relatively high resistance to most antibiotics ranging from 25.0% to 78.6%. The highest rate of resistance was for amoxicillin (n = 11, 78.6%) followed by penicillin (n = 25, 73.5%), sulfamethoxazole/trimethoprim (n = 18, 62.1%), gentamicin (n = 9, 52.9%) and cefuroxime (n = 2, 40%). The resistance to cloxacillin, oxacillin, ofloxacin, erythromycin, tetracycline, and cephalothin ranged between 34.5%–25%. *Staphylococcus* were less resistant to fusidic acid (n = 1, 2.9%). All *staphylococcus* isolates were found to be sensitive to vancomycin and amikacin (Table 5).

Most *E. coli* isolates were resistant to sulfamethoxazole/trimethoprim (n = 18, 85.7%) and amoxicillin (n = 19, 86.4%). *E. Coli* showed resistance to piperacillin/tazobactam, tetracycline, amoxicillin/clavulanic acid, penicillin and erythromycin ranging between 25%–50%. On the other hand, *E. Coli* showed lower rates of resistance to gentamicin (n = 3, 17.65%), cefuroxime (n = 2, 10.5%), cephalothin (n = 1, 10%), and ciprofloxacin (n = 1, 7.7%). All *E. Coli* isolates (100%) were sensitive to ceftriaxone and chloramphenicol (Table 5).

*Klebsiella* isolates were resistant to cephalothin (n = 4, 80.0%), amoxicillin (n = 4, 57.1%), sulfamethoxazole/trimethoprim (n = 4, 57.1%), cefuroxime (n = 2, 33.3%), and piperacillin/tazobactam (n = 1, 33.3%). All *Klebsiella* (n = 5, 100%) showed sensitivity to ciprofloxacin. However, the ESBL-*K. pneumonia* isolates (n = 7) were resistant to nearly all commonly used antibiotics such as amoxicillin, ceftriaxone, cefuroxime, gentamicin, and sulfamethoxazole/trimethoprim (Table 5).

## Discussion

The study was aimed at determining the antimicrobial sensitivity patterns of common microorganisms isolated from clinical samples of CSF and to recommend appropriate medicines for the empirical treatment of meningitis in Namibia.

There are several laboratory tests that are useful in the diagnosis of central nervous system infection, yet no single laboratory test or clinical feature can distinguish between different types of central nervous system infections. Some clinicians propose clinical decision rules which combine clinical and simple laboratory features. However, microbial culture and identification remain the gold standard for diagnosing bacterial meningitis [11,12].

The etiology of central nervous system infections differs from place to place and with different age groups [13,14]. Studies have shown that bacterial meningitis is responsible for about 30%– 40% of central nervous system infections. The remaining 60-70% are due to other etiologies such as viral meningitis, cryptococcal meningitis, brain abscess, tuberculosis meningitis, tuberculoma and others [13,15].

In this analysis, 9.6% of the CSF samples showed microorganism growth. Studies have shown that CSF cultures are expected to be positive in 70%–85% of patients with bacterial meningitis who had no prior antimicrobial therapy [4,15,16]. In the present study, prior antibiotic treatment and high aseptic meningitis associated with HIV may have contributed to the low bacteria growth [4,16]. Autoimmune processes, HIV itself, and meningitis caused by fungal infections that occur in patients with late stage HIV infection can present with signs and symptom of meningitis with negative CSF culture [17].

The most common pathogens causing meningitis that were isolated from the CSF samples analysed by NIP were *Streptococcus species, N. meningitidis*, *Haemophilus influenzae*, *Staphylococcus,* and *E. coli*. All common organisms isolated from CSF the samples showed high resistance to penicillin. The 34.3% resistance of *Streptococcus species* to penicillin is much higher than the 24.8% resistance in the United States [18]. This is surprising as resistance rates are generally expected to be higher in developed countries [19].

The sensitivity to cephalosporins remained high for *Streptococcus*, *Neisseria*, *E. coli,* and *Haemophilus*. The 96.4% sensitivity of *S. pneumoniae* for cefuroxime was comparable with 91.4% sensitivity in the United States [20] The highest percentage of resistance to a cephalosporin was seen among ESBL *K. pneumoniae*, *Klebsiella*, and *Staphylococcus*.

The profile of isolated organisms differed by age group. *Staphylococcus* seemed to be the common cause of meningitis in all age groups. *Staphylococcus* was moderately sensitive (60%–75%) to the cephalosporins (cefuroxime, cephalothin) but it showed less sensitivity to penicillins (23.5%) and zero sensitivity to sulfamethoxazole/trimethoprim which is not used in the empirical treatment of patients with suspected meningitis.

ESBL *K. pneumoniae* was the most frequent isolate in CSF samples drawn from neonates (4/9). This finding is different from the known common pathogens that cause meningitis during the first week of life in the United States [4]. There has been a significant increase in ESBL *Klebsiella species* reported in recent years [21,22]. These strains are highly virulent and have an extraordinary ability to spread, and can result in bacteremia and significantly increase mortality. *Klebsiellae* have been incriminated in nosocomial infections [21,22]. ESBL *Klebsiella* has become a major problem in hospitals because of resistance to multiple antibiotics [22]. In addition to meningitis, it can cause pneumonia, bacteremia, thrombophlebitis, urinary tract infection, cholecystitis, diarrhea, upper respiratory tract infection, wound infection, osteomyelitis, and meningitis.

The medical use of invasive devices, contamination of respiratory support equipment and use of antibiotics are factors that increase the likelihood of nosocomial infection with *Klebsiella species*. Sepsis and septic shock may follow entry of organisms into the blood from a focal source. The Namibia STGs recommend empirical treatment of suspected meningitis in children younger than three months with amoxicillin combined with gentamicin as first choice and ceftriaxone as alternative. Yet, the *ESBL’s Klebsiella* isolate was found to be sensitive to ciprofloxacin, imipenem, and piperacillin/tazobactam and resistant to almost all the medicines included in the Namibia STGs, such as amoxicillin and gentamicin.

Most of the ESBL *K. pneumoniae* were isolated from CSF samples drawn from neonates. *Haemophilus* species were common isolate in one month to 11 months and 1–5 years age groups. *Haemophilus, Streptococcus,* and *Staphylococcus* were common in CSF samples drawn from infants aged from one month to 11 months whereas *Streptococcus, Neisseria,* and *Haemophilus* were commonly isolated in the 1–5 years age group. In the 6–12 years age group, *Neisseria* and *Streptococcus* were the most common isolate. *Streptococcus pneumoniae, Neisseria specie, Escherichia coli and Staphylococcus* were common isolate in adults. *Streptococcus pneumoniae and Neisseria specie* were common isolate in all age group and were found to be sensitive to cephalosporins recommended in the STGs (ceftriaxone and cefuroxime). However, 40.0% of *Staphylococci* and 25.0% of *Haemophilus* isolates were resistant to cefuroxime. *Staphylococcus* and *Haemophilus* species were also found to be resistant to amoxicillin in 78.6% and 34.0% of the cases. *Staphylococci* and *Haemophilus* were second and third common isolate in infants aged one month to 11 months following *Streptococcus pneumoniae*. Other studies have indicated that 80% of community-acquired bacterial meningitis in children aged three months and older are due to *S. pneumoniae* and *N. meningitidis* and empirical coverage with cephalosporin (cefuroxime or ceftriaxone) is recommended [10].

In adults and adolescents, *Streptococcus* and *Neisseria* were the most frequent isolates—this is consistent with the finding of other similar studies [10]. Both have high sensitivity to ceftriaxone.

### Strengths and implications

The strength of this epidemiologic analysis is that it provided the national picture of microorganisms causing CNS infection and their resistance pattern. It also showed the common isolate among different age group and their resistance pattern. These finding can be used to guide empirical treatment of patients with suspected meningitis. It can also inform the review of treatment guidelines. This exercise demonstrated the importance of analyzing routinely collected clinical laboratory data in the monitoring of the emergence of antimicrobial resistance. The analysis was time efficient and inexpensive. The analysis can be replicated on other clinical conditions to identify the common causative organism or repeated at a later date to see trends in antimicrobial resistance patterns.

## Limitations

The main limitation of this analysis was the use of secondary data. The data was not primarily collected to answer a specific *a priori* research question but as part of NIP’s routine testing clinical samples. The laboratory tests’ results are recorded mainly for reporting back to clinicians as well for administrative and billing purposes. As a result of this set up, not all species of microbes were identified or tested against the antibiotics of interest. There were also some important variables not captured in the system, including the possible alternative diagnoses and a patient’s history of prior treatment with antibiotics before collection the CSF samples.

## Conclusions

The common organisms isolated from CSF samples submitted to the NIP were *Streptococcus Pneumoniae*, *Neisseria meningitidis*, *Haemophilus influenzae*, *Staphylococcus*, and *E. coli*. All common organisms isolated from CSF showed high sensitivity to the cephalosporins used in the empirical treatment of meningitis in Namibia. The resistance of common isolates to penicillin which is alternative to cephalosporins in the STG is high*.*

Most ESBL *K. pneumoniae* isolated from CSF samples were from neonates and were found to be resistant to the antibiotics recommended in the Namibia STGs for treating meningitis in children aged three months or younger. Based on the above findings, it is recommended to use a combination of aminoglycoside and third-generation cephalosporin to treat non–ESBL *Klebsiella* isolates. Carbapenems (e.g., meropenem) and piperacillin/tazobactam should be considered in severely ill patients with possible ESBL *Klebsiella* infection.

There is a need to strengthen the infection control practices in the public hospitals in Namibia. It is also recommended that a national antimicrobial resistance surveillance system be developed for early detection of resistance. This would be expected to help identify appropriate antibiotics for the management of meningitis in Namibia.

## Competing interests

None of the authors have any competing interests to disclose.

## Authors’ contributions

AM analyzed the data, interpreted the results, and drafted and finalized the manuscript. GU and CN assisted in extracting and cleaning the data. JG, DM, FK, and CN reviewed the protocol for the analysis and the final draft of the manuscript. KK, LI, and MPJ reviewed the final draft of the manuscript. ES guided the design of the analysis, provided input into the interpretation of the results, and critically reviewed all drafts of the manuscript. All authors read and approved the final manuscript.

## Declaration

This manuscript is original and the work has not been published elsewhere.
